# The influence of the pharmaceutical industry and the pharmacy sector on Aboriginal and Torres Strait Islander health: a yarning study

**DOI:** 10.1093/heapro/daag115

**Published:** 2026-09-08

**Authors:** Troy Walker (Yorta Yorta), Alessandro C Crocetti, Nichole Lister (Wonnarua), Karen Hill, Simone Sherriff (Wotjobaluk), Jennifer Lacy-Nichols, Yin Paradies (Wakaya), Andrew D Brown, Jennifer Browne

**Affiliations:** Global Centre for Preventative Health and Nutrition, Institute for Health Transformation, School of Health and Social Development, Faculty of Health, Deakin University, 1 Gheringhap Street, Geelong, Victoria 3220, Australia; Global Centre for Preventative Health and Nutrition, Institute for Health Transformation, School of Health and Social Development, Faculty of Health, Deakin University, 1 Gheringhap Street, Geelong, Victoria 3220, Australia; Global Centre for Preventative Health and Nutrition, Institute for Health Transformation, School of Health and Social Development, Faculty of Health, Deakin University, 1 Gheringhap Street, Geelong, Victoria 3220, Australia; NIKERI Institute, Faculty of Arts and Education, Deakin University, 75 Pigdons Road, Waurn Ponds, Victoria 3216, Australia; Global Centre for Preventative Health and Nutrition, Institute for Health Transformation, School of Health and Social Development, Faculty of Health, Deakin University, 1 Gheringhap Street, Geelong, Victoria 3220, Australia; Poche Centre for Indigenous Health, Faculty of Medicine and Health, Edward Ford Building, University of Sydney, Camperdown, New South Wales 2050, Australia; Melbourne School of Population and Global Health, Faculty of Medicine, Dentistry and Health Sciences, University of Melbourne, 207 Bouverie Street, Carlton, Victoria 3053, Australia; School of Humanities and Social Sciences, Faculty of Arts and Education, Deakin University, 221 Burwood Hwy, Burwood, Victoria 3125, Australia; Global Centre for Preventative Health and Nutrition, Institute for Health Transformation, School of Health and Social Development, Faculty of Health, Deakin University, 1 Gheringhap Street, Geelong, Victoria 3220, Australia; Global Centre for Preventative Health and Nutrition, Institute for Health Transformation, School of Health and Social Development, Faculty of Health, Deakin University, 1 Gheringhap Street, Geelong, Victoria 3220, Australia

**Keywords:** commercial determinants of health, First Nations health, Aboriginal and Torres Strait Islander health, pharmaceutical industry, pharmacy sector

## Abstract

While pharmaceutical companies develop life-saving medicines and the pharmacy sector is integral to healthcare delivery, entities within the medicines sector can also operate as powerful commercial actors. The practices of the pharmaceutical industry and the pharmacy sector warrant scrutiny through a commercial determinants of health lens. This is particularly important for Indigenous peoples, who experience inequities within Western healthcare systems while also holding enduring cultural knowledge of traditional medicines. Despite increasing international attention to the practices of the pharmaceutical industry, the influence of the medicines sector, as a whole, on Aboriginal and Torres Strait Islander peoples in Australia has not previously been examined. This study aimed to examine the systems, practices and pathways through which the pharmaceutical industry and the pharmacy sector may influence Aboriginal and Torres Strait Islander health and wellbeing. We applied the Aboriginal research method of Yarning to explore the perspectives and experiences of ten Aboriginal/Torres Strait Islander and nine non-Indigenous participants with expertise relevant to the pharmaceutical industry and/or the pharmacy sector. Five themes revealed tensions between commercial models within the pharmacy sector and community needs; regulatory marginalization of cultural medicines and the need to protect Indigenous cultural and intellectual property; pharmaceutical industry efforts to partner with Aboriginal organizations; the lobbying power of peak pharmacy sector bodies; and opportunities for genuine collaboration. Strengthening culturally safe practice, embedding pharmacists within Aboriginal Community Controlled Health Services, and elevating Aboriginal and Torres Strait Islander governance across the medicines sector are critical to addressing power imbalances and advancing health equity.

Contribution to Health Promotion

- This study provides new insight into how the pharmaceutical industry and pharmacy sector may influence Aboriginal and Torres Strait Islander health.
- Indigenous knowledges, cultural and intellectual property and cultural medicines are inadequately recognized within current medicines policy and regulatory systems.
- Some medicines sector actors employ corporate social responsibility practices to engage with Aboriginal and Torres Strait Islander communities, despite lobbying that conflicts with community-controlled health priorities.
- There may be potential for genuine collaboration, particularly with the pharmacy sector; however, Aboriginal and Torres Strait Islander leadership and governance are essential.

## Introduction

The pharmaceutical industry and the pharmacy sector, together forming the medicines sector, have essential roles in healthcare. Yet the practices of commercial actors within this sector are increasingly recognized as commercial determinants of health (CDoH). CDoH are defined as ‘the systems, practices, and pathways through which commercial actors drive health and equity’, recognizing that diverse commercial entities can influence health both positively and negatively (Gilmore *et al*. 2023). Within the medicines sector, the pharmaceutical industry comprises large multinational corporations involved in the research, manufacture, marketing, and distribution of medicines. Global spending on medicines was projected to reach US$1.6 trillion in 2025, underscoring the pharmaceutical industry’s economic scale and market power (IQVIA 2021). In Australia, the pharmacy sector, comprising wholesalers, retail pharmacy chains, and community pharmacies, operates at the intersection of healthcare and business, and is increasingly concentrated, with market share growing among a small number of large companies (Australian Competition and Consumer Commission 2024). While actors within the medicines sector, including community pharmacists, pharmacy owners, pharmaceutical manufacturers and wholesalers, and their representative bodies, are diverse and sometimes pursue conflicting interests, they collectively shape access to medicines. Understanding their collective systems and practices through a CDoH lens is important for identifying how commercial practices shape health outcomes, particularly for Aboriginal and Torres Strait Islander peoples.

The pharmacy sector encompasses two distinct but overlapping interests: healthcare professionals and retail and community pharmacies. Pharmacists are registered healthcare professionals responsible for dispensing medicines, providing medication reviews, patient education and clinical advice, represented nationally by the Pharmaceutical Society of Australia (PSA). Retail and community pharmacies, ranging from large chains to independently-owned businesses, predominantly operate within a commercial model to dispense medications, market and sell other products, with pharmacy owners’ interests represented by the Pharmacy Guild of Australia (Grattan Institute 2026). Recognizing this distinction is important because pharmacists employed within profit-driven pharmacy models may face tensions between their responsibilities as health professionals and the commercial interests of the private businesses in which they work (Hagsten *et al*. 2024).

Unlike inherently harmful industries such as tobacco and alcohol, the medicines sector provides essential services including development, distribution, and dispensing of life-saving medicines. Yet the pharmaceutical industry and the pharmacy sector often operate through profit-driven commercial systems that can undermine health equity. This dual character distinguishes the medicines sector from established harmful commodity industries and requires a more nuanced CDoH analysis (Thomas *et al*. 2024). On one hand, the sector facilitates access to essential medicines and vaccines; on the other, numerous harmful practices of pharmaceutical companies have been documented in the literature, including price inflation, profiteering, inappropriate marketing, manipulation of research, and payments to doctors and patient organizations (Cutler 2020, Marks 2020, Hassan *et al*. 2021, Tenni *et al*. 2022, Mor *et al*. 2024). This complexity is further shaped by the highly regulated environments in which these actors operate, and by their diverse relationships with governments, health professionals, communities, and other public-facing groups (Hennessy *et al*. 2024, Hooimeyer *et al*. 2024, Randle *et al*. 2024). These systems and practices can ultimately undermine access to medicines and drive health inequities, making CDoH analysis of the medicines sector especially important for Aboriginal and Torres Strait Islander peoples, who experience significant inequities in Australia’s health system.

Aboriginal and Torres Strait Islander peoples, the First Peoples of Australia, have a holistic view of health incorporating physical, social, emotional, cultural, spiritual and ecological dimensions (Verbunt *et al*. 2021). Cultural medicines and healing practices have been maintained for over 65 000 years and represent that world’s oldest continuing medicine systems (Gall *et al*. 2025). Today, Aboriginal and Torres Strait Islander peoples experience the ongoing impacts of colonization, contributing to disparities in chronic and communicable disease, as well as mental health conditions (Australian Institute of Health and Welfare 2024). Despite the disproportionate burden of disease, Aboriginal and Torres Strait Islander peoples experience systemic barriers to accessing health services, prescription medication, and cultural medicines (Gall *et al*. 2025). Responding to healthcare inequities, Aboriginal Community Controlled Health Organisations (ACCHOs) first emerged in the 1970s and now exist throughout Australia delivering culturally responsive comprehensive primary health care for Aboriginal and Torres Strait Islander peoples.

Within this context, recent reforms within the medicines sector are likely to shape how Aboriginal and Torres Strait Islander peoples access medicines, with implications for the pharmaceutical industry, the pharmacy sector, and ACCHOs. For example, Pharmaceutical Benefit Scheme (PBS) listing and pricing decisions determine medicine affordability, while 60-day dispensing increases the interval between prescription refills at a pharmacy, reducing burdens linked to access. Within the pharmacy sector, expanded scope of practice for pharmacists enables retail and community pharmacies to provide a broader range of primary health services, potentially overlapping with those provided by ACCHOs (Cross *et al*. 2024, Pharmacy Guild of Australia 2025).

These developments illustrate how the pharmaceutical industry and the pharmacy sector are interdependent components of the medicines access pathway. Their combined influence as a commercial determinant of health for Aboriginal and Torres Strait Islander peoples has not previously been examined. While earlier research has explored Aboriginal experiences and perspectives related to medication access, adherence, vaccination uptake, and integrating pharmacists within ACCHOs (Couzos *et al*. 2011, 2020, Graham *et al*. 2022), the views of Aboriginal and Torres Strait Islander health professionals and others working in this field regarding the pharmaceutical industry and the pharmacy sector remain largely unexplored, a knowledge gap our ACCHO partners have identified as a priority (Cubillo *et al*. 2025).

The aim of this study was to explore the perspectives of Aboriginal and Torres Strait Islander peoples and non-Indigenous people working in Aboriginal health regarding how the medicines sector influences Aboriginal and Torres Strait Islander health and wellbeing. Our study sought to answer the following question: What are the systems, practices and pathways through which the pharmaceutical industry and the pharmacy sector may be influencing Aboriginal and Torres Strait Islander health and wellbeing?

## Materials and methods

### Researcher positionality

This study was led by a Yorta Yorta (Aboriginal) clinician and researcher (T.W.) working alongside Aboriginal (N.L., Y.P., S.S.) and non-Indigenous (A.C.C., K.H., J.B., A.D.B., J.L.-N.) researchers with expertise in health policy, public health, systems science, CDoH, Aboriginal health and nutrition. The research question was conceived by T.W. drawing on his clinical work in Aboriginal communities and lived experiences as a Yorta Yorta man and further refined in response to community priorities and inputs from ACCHO partner organizations and their members. We (T.W., N.L., J.B.) determined that the CONsolIDated critERia for strengthening the reporting of health research involving Indigenous Peoples statement, developed by Huria and colleagues, was ideally placed to guide the reporting of this research in a manner honouring cultural ‘right way’ (Huria *et al*. 2019, O’Donnell-Barber and Menzel 2025). The checklist contains seventeen items to strengthen research practices and dissemination of research with Indigenous peoples.

### Governance

The study forms part of a broader, Aboriginal-led programme of research on the commercial determinants of Aboriginal and Torres Strait Islander health, undertaken in partnership with national, state, and territory peak bodies in the ACCHO sector (Cubillo *et al*. 2025). To uphold the right to self-determination, safeguard Indigenous cultural and intellectual property, and ensure the research reflected Aboriginal and Torres Strait Islander priorities and values, a governance model was developed that prioritized Indigenous data sovereignty, community and ACCHO engagement, capacity building, and knowledge translation (Maiam nayri Wingara 2018, Cubillo *et al*. 2025). The governance structure included an ACCHO Circle, comprising representatives from partner organizations and an Indigenous Data Governance group who provided input and approved the study protocol, data management plan and research outputs.

### Study design

We used the Aboriginal research methodology of Yarning to explore domains of enquiry around the influence of both pharmaceutical industry and pharmacy sector actors on Aboriginal and Torres Strait Islander health. The study design, collection, analysis, and interpretation of data were embedded in an Indigenous research paradigm outlined by Kennedy and colleagues (Wilson 2014, Kennedy *et al*. 2022), alongside frameworks on the CDoH (Legg *et al*. 2021, Gilmore *et al*. 2023).

### Participant selection and recruitment

Purposive sampling was initially undertaken, guided by our governance groups, to recruit participants with the expertise best suited to discussing the research question. In selecting participants, we aimed to include Aboriginal and Torres Strait Islander and non-Indigenous peoples from a range of professional backgrounds, including public health research, health promotion advocacy, the ACCHO sector, community pharmacy, and government, who held relevant professional knowledge or experience with the pharmaceutical industry and/or pharmacy sector. We also utilized snowball sampling, whereby participants nominated other potential participants. All participants worked within the health sector and had expertise directly relevant to the medicines sector resulting from their roles in community pharmacy, ACCHOs, public health, or government-based positions. Potential participants were contacted by email and those who were interested or requested further information were offered an online preparatory conversation with the lead investigator (T.W.). Participants were provided with a plain language statement, including information about the study and the option to withdraw at any time. Through this approach, 22 participants were invited and nineteen were recruited based on study timeframes and availability.

### Data collection

We used a Yarning approach to explore domains of enquiry around perspectives and lived experiences related to the medicines sector (Bessarab and Ng'andu 2018, Hunter 2023). A Yarning approach allowed for respectful and authentic collaboration with participants by privileging Indigenous knowledges, positioning participants as experts and knowledge holders in their fields of lived experience; embedding respectful exchange through Yarning; creating opportunities for sustained relationships with researchers; and assuring participants that findings would be shared in accessible, respectful, and sensitive ways that align with community priorities (Bessarab and Ng'andu 2018, Kennedy *et al*. 2022, Hunter 2023, Rowley *et al*. 2025).

Yarns took place between October 2024 and July 2025 using MS Teams or Zoom videoconferencing software following informed written and verbal consent. Participants were asked to share their views on the practices of the pharmaceutical industry and pharmacy sector and how these influence Aboriginal and Torres Strait Islander health (Table 1). Participants were encouraged to share their views and experiences, whether they were positive, negative, or neutral.

**Table 1:** Yarning guide.

Domain	Examples of topics and questions guiding yarns
Social yarning (not recorded)	Introductions Social and cultural connections Study focus and purpose of yarn Confirmation of consent and permission to record
Participant background	Can you tell me a little about yourself and your past and present work experiences?
Research topic yarning	What are your initial thoughts when you think of either the pharmacy sector or the pharmaceutical industry and their impact on Aboriginal and Torres Strait Islander people?
	What do you think they are doing that may affect the health of Aboriginal and Torres Strait Islander people?
Pharmacy sector representative bodies	Have you heard of some of the key pharmacy sector organisations in Australia, including the Pharmacy Guild of Australia (PGA) and the Pharmaceutical Society of Australia (PSA)?
	What do you think about these organisations having Reconciliation Action Plans (RAPs)?
Lobbying and policy influence	Can you share any examples you know about where pharmacy or pharmaceutical bodies lobbied the government or attempted to influence policy?
	The federal government recently extended prescriptions for certain medications dispensed at pharmacies from 30 to 60 days. What effect do you think this will have on health and wellbeing for Aboriginal people?
Corporate social responsibility	What are your thoughts on pharmaceutical industry funding for health research, health professional training, conference attendance or scholarships?
Marketing	Can you tell me about any pharmacy or pharmaceutical marketing you've seen, including online, on television, social media, or in medical journals?
Solutions and policy implications	What do you think government should do to address the issues we have discussed today?
Closing	Issues not already covered Key messages participants would like to end with.

Yarns were conducted individually with most lasting around 1 h and durations ranging between 40 and 90 min. They were recorded, with permission, and audio files were professionally transcribed and deidentified by a third-party provider. Participants were forwarded a copy of their transcript to review and edit if they wished. Of the nineteen participants, four (20%) made minor changes to their original transcript, such as re-wording responses without changing the content or sentiment. One participant requested the removal of one of their responses.

### Data analysis

Yarning transcripts were reflexively analysed, guided by the Reflexive Thematic Analysis method outlined by Braun and Clarke and adapted by Kennedy and colleagues (Braun and Clarke 2021, Kennedy *et al*. 2022). After listening to recordings, each transcript was coded by T.W. to identify initial patterns in the data. Coding occurred inductively with active consideration of existing frameworks on the CDoH and other relevant literature (Legg *et al*. 2021, Friel *et al*. 2023, Gilmore *et al*. 2023). Initial codes were mapped into categories and further grouped into preliminary themes using Microsoft Excel following discussion between two authors (T.W., A.C.C.). Preliminary themes were discussed among co-authors (T.W., A.C.C., K.H., N.L., J.L.N.) and refined through ongoing research team collaborative yarns to ensure themes both addressed the research question and appropriately represented participant knowledge. Reflexive note taking and collaborative yarns were central to the analytical process to centre and privilege the voices of each participant. Feedback on preliminary findings was provided to the research team through the project’s governance groups.

### Ethics

This study was conducted according to the National Health and Medical Research Council Ethical Conduct in Research with Aboriginal and Torres Strait Islander Peoples and Communities, and the Aboriginal Health and Medical Research Council (AHMRC) ethical guidelines to ensure culturally appropriate and respectful research involving Aboriginal and Torres Strait Islander communities (National Health Medical Research Council 2018, Aboriginal Health Medical Research Council of NSW 2023). The study was approved by the Deakin University Human Research Ethics Committee (approval no: 2024/HE000248) and the AHMRC Human Research Ethics Committee (approval no: 2335/24). Written informed consent was obtained from all participants.

## Results

### Participant characteristics

Nineteen people participated in the study. All participants had a health background, spanning pharmacy practice (*n* = 4), research (*n* = 5), the ACCHO sector (*n* = 6), government (*n* = 2) and non-government organizations (*n* = 2). Ten participants (53%) identified as Aboriginal and/or Torres Strait Islander, most participants (*n* = 13/19) were female, and the majority (*n* = 16/19) lived in urban locations (Table 2).

**Table 2:** Participant demographics.

Sector	Total	Aboriginal and/or Torres Strait Islander	Non-Indigenous	Male	Female
ACCHO	6	2	4	3	3
Government	2	0	2		2
Corporate	1	1	0	0	1
NGO	2	2	0		2
Research	5	4	1	2	3
Practice	3	1	2	2	1

Five themes were constructed from the data. These covered tensions between commercial models within the pharmacy sector and community interests; accessing cultural medicines while protecting Indigenous cultural and intellectual property; whether medicines sector partnerships are genuine or performative; the lobbying power and influence of the pharmaceutical industry and pharmacy sector representative bodies; and Aboriginal and Torres Strait Islander leadership and genuine collaboration.

#### Theme 1: commercial models within the pharmacy sector versus community interests

Participants highlighted tensions between the commercial imperatives of the pharmacy sector and the holistic models of care prioritized by ACCHOs. Many participants mentioned the underrepresentation of Aboriginal and Torres Strait Islander peoples within the pharmacy workforce and how this affects the cultural safety of service provision. While participants recognized the essential role of pharmacists within the health system, they emphasized the positive impact Aboriginal registered pharmacists could have on the pharmacy sector, including through their ability to re-orient the sector towards a health promotion approach, for example, through ‘pharmacy education sessions with Mob […] so we just have a yarn about medicines in general’. (P18) Participants suggested that embedding pharmacy services within ACCHOs could offer an alternative (and often preferred) model, placing cultural safety and positive health outcomes ahead of profit.

Participants articulated how the commercialization of pharmacy services within retail and community pharmacies undermined access to medicines. The pharmacy location rules, agreed between the Australian Government and the PGA (representing pharmacy owners), set out distance-based criteria for establishing new pharmacies. As one participant described, these rules prevent new pharmacies from being established in close proximity to existing ones, meaning that ‘in NT [the Northern Territory], even though an ACCHO could own a pharmacy, none do’ (P1, ACCHO). This participant noted that the current distance-based provisions favour commercial expansion and profit over community need, directly undermining the viability of ACCHO in-house pharmacies. ACCHO sector participants emphasized the value of embedded pharmacy dispensaries, describing them as ‘the preferred outcome for us, for our sector’ (P17, Aboriginal, ACCHO), but noted that ‘due to politics or whatever reasons, we can’t seem to get the support at the national level’ (P17). These findings highlight how regulatory frameworks, negotiated with pharmacy sector representatives, can limit Aboriginal community control over the delivery of essential health services, as this ACCHO participant explained:

It’s the location rules. Darwin’s essentially saturated from the location rules. So, there’s nowhere you could actually legally build one without a minister letter saying, ‘I’ll let you build this pharmacy two doors down from the other pharmacy (P1, ACCHO).

Building on these regulatory tensions, participants described how the commercial orientation of retail and community pharmacies commodified medicines and pharmacy services, positioning them as sources of profit rather than as comprehensive primary health care, particularly for Aboriginal and Torres Strait Islander peoples. Several participants expressed concern that the prevailing business model of retail and community pharmacies is orientated around maximizing revenue through dispensing fees. As one participant explained, ‘you’re fighting against where most of the money comes from […] the number of prescriptions that you process and, yes, the fees that come off of them*’* (P7, Aboriginal Academic).

#### Theme 2: accessing cultural medicines while protecting indigenous cultural and intellectual property

Many participants spoke about the commercial and political barriers impeding access to cultural medicines. One participant explained that ‘there’s a clear message from Mob that they want more recognition, more access to their traditional medicines’ (P12, Academic). Cultural medicines were described by Aboriginal participants as ‘amazingly complex’, ‘synergistically favourable’, and as having ‘symbiotic’ relationships that work based on interconnections of climate, season, temperature and a state of place (P18). However, there was a perception that the ‘western regulation process’ and approval pathways for recognition by the Therapeutic Goods Administration (TGA) were ‘almost insurmountable’ (P12) and heavily influenced by the pharmaceutical industry. This participant suggested that political donations made by the pharmaceutical industry granted them greater access to policy makers and regulatory bodies. They noted that the TGA lacks dialogue ‘with Mob around their medicines’ and should develop ‘a fit-for-purpose entryway for traditional medicines’ informed by Aboriginal and Torres Strait Islander people, rather than the ‘gatekeepers’ within the pharmaceutical industry (P12).

Several participants expressed frustration with what they described as the ‘dominant paradigm’ in Australia which privileges western biomedical knowledge and pharmaceutical science over Indigenous knowledge systems (P8, Aboriginal Academic). As one participant noted ‘there has not been an Aboriginal or Torres Strait Islander traditional medicine that’s been registered’ with the TGA (P12). Participants argued the regulatory and commercial systems in Australia failed to recognize the 65 000 years of cultural medicine practice as an inherent knowledge system that should be respected and strengthened. One Aboriginal pharmacist described the drug approval pathways in Australia as follows:

It is around consumer safety or patient safety. But it doesn’t recognise the evidence of Aboriginal or Torres Strait Islander knowledge systems and how we’ve been able to maintain the evidence of the efficacy of particular medications or the way that they are prepared (P18, Aboriginal pharmacist).

While advocating for the recognition of cultural medicines, some participants expressed concern about the potential appropriation of Indigenous cultural and intellectual property by pharmaceutical companies. One participant perceived that, when evaluating traditional medicines, pharmaceutical scientists would miss the Indigenous ‘value of it’ (P12). Participants emphasized the need to safeguard traditional knowledge from industry exploitation because ‘*t*hey [industry] just use it, they make a shit ton of money off it and we get no benefit’ (P8). This participant recalled a particular interaction with a pharmaceutical industry representative who rationalized novel practices as a benevolent life-saving endeavour, framing Indigenous cultural and intellectual property protection as an impediment to drug development.

The pharmaceutical industry…my impression of them when I was over in Geneva when we were negotiating the treaty negotiations, we had the first ever international law passed that included Indigenous knowledges in intellectual property law. I happened to be sitting in the middle of a bunch of pharmaceutical company reps. It was almost like, for me as an Aboriginal person sitting there who’s doing traditional medicine and all this stuff, it felt like I’d been stuck right in the middle of the enemy. He couldn’t understand why we were wanting to stop the innovation. He believed that us trying to protect Indigenous knowledge would stop the innovation of new drugs because it would just put up too many barriers. (P8, Aboriginal Academic)

#### Theme 3: medicines sector partnerships: genuine engagement or performative social responsibility?

Across participants, a recurring theme was the increasing level of engagement by actors across the medicines sector with Aboriginal organizations and communities. Engagement took different forms, including research funding, scholarships, invitations to sit on advisory committees, Reconciliation Action Plans (RAPs) and other corporate social responsibility initiatives. RAPs are a specific corporate social responsibility document outlining how an organization will contribute to reconciliation with Aboriginal and Torres Strait Islander peoples through relationships, respect, employment, and leadership opportunities (Reconciliation Australia 2025). Currently, RAPs are held by at least sixteen pharmaceutical companies, the peak body representing pharmacy owners, the Pharmacy Guild, and the peak professional body for pharmacists (the PSA), reflecting attempts at engagement across the medicines sector.

Participants expressed mixed views on the credibility of and motives behind medicines sector partnerships. Some described them as genuine commitments to advancing Aboriginal and Torres Strait Islander health, while others characterized them as strategic efforts to secure corporate legitimacy and credibility, the latter being more commonly noted with Aboriginal participants. Participants also shared examples where the pharmaceutical industry provided critical funding support for Aboriginal health research, as one participant recalled, in relation to an ear health study, ‘we were only able to do that trial with the assistance of the pharmaceutical company. They provided the medicines for free’ (P3, ACCHO). Conversely, several participants challenged what they viewed as minimal financial contributions by pharmaceutical companies to Aboriginal and Torres Strait Islander health initiatives, including research and the development of health management resources. One participant characterized such efforts as ‘gammin’ (‘pretend/not true’) reflecting a broader frustration with the tokenistic nature of much industry engagement:

We get pharmaceuticals coming to us all the time saying ‘can we give you ten grand to do a project?’ Now, for me, ten grand is not a lot […] and that amount of money that you’re saying that they’re making in profits is nothing. It is like ten cents to them. You know what I mean? So here they are chucking this little bit […] It’s gammin. (P19, Aboriginal and Torres Strait Islander, NGO)

Partnerships between pharmaceutical companies and pharmacy sector actors and Aboriginal organizations were discussed with caution. Participants from the ACCHO sector noted there may be ‘a willingness from those big corporates to work in collaboration’ (P17) while acknowledging they were not naïve to the fact that ‘pharmaceutical companies, like community pharmacies, are there to make money’ (P1). Some participants questioned the ethics and accountability of such arrangements. As one pharmacy researcher remarked, ‘it’d be great if we tried to limit it as much as possible’, and went on to ask, ‘are there safeguards and accountability mechanisms in place to manage conflicts?’ (P5, Academic).

Participants also expressed nuanced views regarding scholarships for Aboriginal and Torres Strait Islander students funded by commercial actors. Some acknowledged the pragmatic necessity of such support, given the lack of alternative funding sources. As one participant noted, ‘where else are those students going to get that amount of money from?’ (P11, Government). This view was echoed by others, who argued that pharmaceutical companies ‘should be shelling out to support Aboriginal and Torres Strait Islander peoples’ (P14, ACCHO). However, concerns were also raised about the expectations placed on scholarship holders. One participant described the pressure to remain within programmes due to perceived obligations, stating ‘there’s a cultural load that says that if you’ve got a scholarship for X thousand dollars a year, you’ve got to keep going with that for all the people who didn’t get it’ (P15).

#### Theme 4: Power and politics: lobbying and policy influence

The ‘unique relationships’ (P3) between pharmaceutical industry and pharmacy sector representative bodies and the Australian government, including on decisions affecting Aboriginal and Torres Strait Islander health, were raised by many participants. Participants expressed concern about commercial influence on policy decisions, through lobbying, meetings with policy makers, political donations, advocacy campaigns, and the ‘revolving door’ between the medicines sector and government. Participants reflected on both the pharmaceutical industry and the pharmacy sector’s access to and influence over health policy decisions affecting Aboriginal and Torres Strait Islander peoples noting, ‘they’ve got the ear of decision makers’ (P12). Another participant remarked ‘I used to think that policies were written by politicians because it sounded similar. That was very wrong’ (P8). Regarding the ‘revolving door’, one participant mentioned a prominent pharmacy sector lobbyist who had previously ‘been involved in politics’ (P1, ACCHO). The lobbying power of both the pharmaceutical industry and the Pharmacy Guild, as a peak representative body, was frequently described, as illustrated in the following quotes:

I mean the Pharmacy [Guild], they’re up in Parliament House all the time. They have people paid specifically to do that. And so do many of the pharmaceutical companies. (P11, Government).

The funds that the [Pharmacy] Guild have and pharmaceutical [companies] have is massive and so when that capital is being mobilised by non-Indigenous people, it’s going to go towards things that aren’t necessarily […] going to benefit Aboriginal and Torres Strait Islander people (P1, ACCHO).

A key example shared by participants was the Eighth Community Pharmacy Agreement, a 5-year funding and regulatory agreement between the Australian Government and the Pharmacy Guild that governs the operation of community pharmacies. Participants described the Pharmacy Guild as highly skilled and active in lobbying, representing the interests of the pharmacy owners, while negotiating agreements which directly affect access to medicines for Aboriginal and Torres Strait Islander peoples. Another participant with experience in health policy and politics noted that ‘the Pharmacy Guild gets a lot of money through the [Community Pharmacy] Agreements that’s supposed to be about delivering primary care services’ (P11, Government).

Another example participants provided of lobbying was during the introduction of the 60-day dispensing laws in Australia. This policy allows patients to receive a 2-month supply of eligible medicines, effectively halving dispensing fees. ACCHO sector participants supported the initiative, describing it as ‘a great thing’ (P3) that would benefit Aboriginal and Torres Strait Islander peoples, particularly those in rural and remote areas, by reducing travel expenses and out-of-pocket costs. In contrast, participants reported that the Pharmacy Guild strongly opposed the reform, arguing it would threaten the financial viability of retail and community pharmacies. Participants from the pharmacy sector expressed concerns about potential revenue losses, medication supply shortages, and reduced ‘opportunities in an extra month of not engaging with your regular pharmacist’ (P16). Ultimately, the 60-day dispensing policy was introduced, prompting an early renegotiation of the Community Pharmacy Agreement with additional funding guaranteed for retail and community pharmacies, an outcome one participant reflected ‘just speaks to the power of the pharmacy lobby movement’ (P1).

#### Theme 5: Aboriginal and Torres Strait Islander leadership and genuine collaboration

In contrast to the concerns outlined in the previous sections, several participants identified promising developments and opportunities for culturally safe pharmacy practice in Aboriginal and Torres Strait Islander contexts. Aboriginal pharmacists highlighted the value of partnership the PSA, the professional body representing pharmacists, as distinct from the Pharmacy Guild, representing the business interests of pharmacy owners. One example of effective partnership between the pharmacy profession and ACCHO sector noted by participants was the Deadly Pharmacists Foundations Training Course, a free, seven-module online training programme, co-created by the PSA and the National Aboriginal Community Controlled Health Organisation (NACCHO), to equip pharmacists with the skills they need to work effectively within ACCHOs. Aboriginal pharmacists praised the course for its focus on culturally safe care within the pharmacy sector, content largely absent from Western university curricula or profit-driven retail and community pharmacies, where pharmacists ‘would never have really got this sort of education’ (P7) and for the addition of new modules ‘specifically developed for mental health and working with Aboriginal and Torres Strait Islander people in a pharmacy setting’ (P18). This initiative was cited as a leading example of how peak bodies from the ACCHO and pharmacy profession can work collaboratively to advance health equity.

Advancing Aboriginal and Torres Strait Islander leadership in the medicines sector was highlighted by many participants as a key strategy to ‘honour and respect community control’ (Participant 6, Aboriginal, ACCHO), and countering for-profit approaches. Participants from the ACCHO sector suggested increasing the number of qualified Aboriginal pharmacists and ‘training of Aboriginal health workers and practitioners’ in quality use of medicines (P6) to create an Aboriginal pharmacy workforce ‘that will provide that leadership, culturally safe care and representation for the sector’ (P1). Integrating pharmacists into comprehensive primary health care teams within ACCHOs, following successful trials in several Australian jurisdictions, was identified by many participants as a key aspect of culturally safe service models. ACCHO sector participants advocated for this approach, while noting the peak body for pharmacy owners, ‘the Pharmacy Guild of Australia did not support the model […] because it takes pharmacists out of community pharmacy’ (P3). As one Aboriginal pharmacist explained, embedding pharmacists within ACCHOs offers far more than the ‘convenience’ (P18) of on-site dispensing, it reframes their role from a business transaction to an essential health professional:

The role of a pharmacist in an ACCHO is to use their skills and knowledges in a multidisciplinary team to make sure that when the patient sees the GP, that the medications have been reviewed […] it’s actually a synergistic relationship that has the opportunity to produce better health outcomes (P18, Aboriginal Pharmacist).

Another priority expressed by participants for enhancing the health promoting impacts and preventing the potential negative impacts of practices within the medicines sector was to strengthen Aboriginal and Torres Strait Islander leadership at the policy level. As one participant put it, priority should go to getting ‘First Nations mob around some of those decision tables’ (P17). Participants advocated positioning Aboriginal and Torres Strait Islander peoples in senior roles in both the public and private sector to ensure greater transparency and input into policy and funding decisions. This included decisions about listing specific drugs on the PBS so Aboriginal and Torres Strait Islander peoples can access them at subsidized rates. As one participant explained, the current system is ‘market-based’ relying on ‘a company who [is] actually willing to make that available and put it through the PBS process’ (P1). Within the pharmaceutical industry, a key recommendation was to ensure ongoing Aboriginal and Torres Strait Islander leadership in the governance of cultural medicines, addressing the ‘“issues we keep coming up against” regarding Indigenous Cultural and Intellectual Property and who owns that knowledge […] from our perspective, as Aboriginal people, no one owns it. Country owns it’ (P8). Some participants expressed that the pharmaceutical industry has an obligation to provide reciprocal support to Aboriginal and Torres Strait Islander peoples and the ACCHO sector, given that some of their profits are derived from the disproportionate burden of diseases requiring prescription medications.

It’s time for the big corporates and the business sector to also assist because, let’s face it, they receive huge funding injections from the Aboriginal sector. All the services—well for example the pharmaceutical sector. I mean, they get huge injections from the Aboriginal community-controlled sector, purely from the medication that our Mob need (P17, Aboriginal, ACCHO).

## Discussion

To our knowledge, this is the first study exploring the perspectives of Aboriginal and Torres Strait Islander and non-Indigenous participants across academic, pharmacy, ACCHO, government, and non-government organization settings about the influence of the pharmaceutical industry and the pharmacy sector on Aboriginal and Torres Strait Islander health and wellbeing. Our findings provide novel insight into participants’ perspectives on the tensions between current pharmacy sector practices and ACCHO models of care; the marginalization of Indigenous knowledges within western regulatory systems; the challenge of distinguishing between genuine partnerships and performative reputation management; the lobbying power and influence of the pharmaceutical industry and the Pharmacy Guild; and the importance of Aboriginal and Torres Strait Islander leadership and genuine collaboration. From a CDoH perspective, this study positions multinational pharmaceutical companies and commercial actors within the pharmacy sector as corporate entities embedded within profit-maximizing systems, while recognizing that pharmacists, as health professionals within the medicines sector, may be both shaped by and resistant to these commercial pressures. Together, these findings offer a nuanced understanding of how the systems, practices, and pathways of the medicines sector may influence Aboriginal and Torres Strait Islander health. These are elaborated below.

At the systems level, our findings illustrate how political, economic, and regulatory settings across the medicines supply chain tend to favour commercial interests over the priorities of Aboriginal organizations and communities. Participants in our study echoed previous research emphasizing the importance of cultural medicines in supporting holistic health, wellbeing, and cultural continuity. Yet Indigenous knowledges remain poorly recognized in current medicines policy and western regulatory systems (Gall *et al*. 2018, 2025, Williams *et al*. 2023). As of 2025, no Aboriginal or Torres Strait Islander cultural medicines have been listed or registered by the TGA, reflecting how regulatory frameworks can reproduce colonial governance structures (Marshall *et al*. 2024). The Science for Profit Model provides a useful lens for understanding these systemic issues, examining the strategies commercial actors deploy to influence science and policy decision-making in ways that serve their interests (Legg *et al*. 2021). Large multinational pharmaceutical companies, through their scientific practices and commercial strategies, influence drug development and approval pathways, marginalizing cultural knowledges, or appropriating them for commercial gain (Legg *et al*. 2021, Lal *et al*. 2024, Redvers *et al*. 2025). Although international agencies, such as the World Health Organization and the World Intellectual Property Organization, recognize the rights of Indigenous peoples to protect their traditional medical knowledge, Australia is yet to implement laws to prevent misappropriation of Indigenous cultural and intellectual property (World Intellectual Property Organization 2023, Marshall *et al*. 2024, World Health Organization 2025). Importantly, TGA approval and Pharmaceutical Benefits Scheme listing of cultural medicines would represent a fundamentally different non-commercial model of medicines access. However, whether and how current regulatory frameworks could be adapted to recognize Indigenous knowledge systems is an important question for future research. These challenges are compounded by pharmaceutical company influence on Australia’s medicines regulatory environment, including decision-making within the TGA and the Pharmaceutical Benefits Scheme, the agencies empowered to approve and fund cultural medicines (Vaughan 1995, Yoffe *et al*. 2023, Gall *et al*. 2025).

Our findings also point to a system-level misalignment between pharmacy sector service models and ACCHO sector priorities. The Australian pharmacy sector is dominated by retail and community pharmacies operating within a commercial environment, with the Pharmacy Guild representing the interests of pharmacy owners in policy development (Grattan Institute 2017, 2026). In contrast, ACCHOs deliver comprehensive, culturally safe primary health care, grounded in the principles of community control, self-determination, and a holistic view of health (Mackean *et al*. 2025). The present study extends critiques of the corporatization of healthcare in Australia by highlighting how Pharmacy Guild policy positions can be at odds with ACCHO priorities, including efforts to embed dispensaries and pharmacists into ACCHO primary health care teams (Docking *et al*. 2025), a misalignment identified in previous research highlighting inadequate system support for developing effective relationships between ACCHOs and community pharmacists (Swain and Barclay 2015, Drovandi *et al*. 2022). Public health advocates have argued that Community Pharmacy Agreement negotiations between the Pharmacy Guild and the Australian Government lack transparency and serve the interests of pharmacy owners rather than consumers (Grattan Institute 2017, 2026), a concern illustrated by the scale of the Eighth Community Pharmacy Agreement. The agreement committed $26.5 billion in funding to retail and community pharmacies over 5 years (Department of Health and Aged Care 2024), a 22% increase on the previous agreement according to the Pharmacy Guild (Pharmacy Guild of Australia 2024).

The pharmacy location rules illustrate how this system-level misalignment is institutionalized in policy. Negotiated between the Australian Government and the Pharmacy Guild, the rules set out distance-based criteria and commercial co-location requirements for establishing new pharmacies, with no exemption pathways for ACCHOs (QAIHC 2024, Department of Health Disability and Ageing 2025, Grattan Institute 2026). While the rules nominally aim to ensure equitable, timely, and affordable access to medicines, their provisions, including distance criteria designed around commercial retail environments (Department of Health Disability and Ageing 2025), structurally restrict ACCHOs from operating their own pharmacies, despite evidence that medication dispensaries within ACCHOs improve medication adherence and access for Aboriginal and Torres Strait Islander peoples, particularly in remote areas and for those who have had negative experiences within the mainstream health sector (Couzos 2005, Couzos *et al*. 2020).

At the level of commercial practices, our findings highlight lobbying and policy influence by pharmaceutical industry and pharmacy sector actors as key mechanisms through which commercial interests may be prioritized over Aboriginal and Torres Strait Islander health priorities. Creating industry-friendly policymaking environments is a well-documented strategy used by commercial actors, including the pharmaceutical industry, to shape policy decision-making in the industry’s favour (Legg *et al*. 2021). The Pharmacy Guild donated over $600 000 to political parties in 2024–25, with individual pharmaceutical companies and Medicines Australia making additional contributions, together totalling over $1.1 million from the medicines sector in an election year (Australian Electoral Commission 2026). The Pharmacy Guild demonstrated its lobbying capacity, in 2023, through an intensive campaign against the 60-day dispensing policy reform, despite the ACCHO sector and multiple peak health bodies supporting the change on the basis it would reduce costs and improve access to medicines, especially for Aboriginal and Torres Strait Islander peoples in rural and remote areas (NACCHO 2023). The Pharmacy Guild argued that 60-day dispensing would force 665 pharmacies to close and would result in medication shortages (Pharmacy Guild of Australia 2023, Grattan Institute 2026). Conversely, an independent analysis estimated the policy saved patients over $110 million in its first year of implementation (Grattan Institute 2025). Despite their lobbying activities, the lobbyists working directly for the Pharmacy Guild and the peak pharmaceutical industry body, Medicines Australia, are not covered by Australia’s lobbying code of conduct which applies only to third-party lobbying firms rather than in-house government relations staff (Grattan Institute 2018, Lacy-Nichols *et al*. 2023, Transparency International Australia 2025). Addressing this requires government action to improve transparency about the relationships between commercial actors and policy makers, particularly given Aboriginal community mistrust of both governments and medical institutions (Graham *et al*. 2022). Our findings are consistent with the Science for Profit Model’s identification of political engagement and strategic messaging as key commercial practices shaping policy and regulatory environments (Legg *et al*. 2021).

This study also highlighted how pharmaceutical industry and pharmacy sector actors employ corporate social responsibility practices to engage with Aboriginal and Torres Strait Islander peoples. Participants expressed largely negative perceptions of RAPs and other superficial forms of industry engagement, a finding consistent with other recent work demonstrating RAPs are often viewed by Aboriginal people as tokenistic and transactional, operating as a pseudo-benevolent marketing exercises with vague commitments and weak accountability (Crocetti *et al*. 2024, Leersen *et al*. 2024, 2026). Similarly, we found industry funding for Aboriginal health programmes and scholarships was met with mixed reactions. Some pharmaceutical companies have provided scholarships for Aboriginal and Torres Strait Islander pharmacy students and offered $15 000 scholarships to attend international conferences (NACCHO 2025, National Indigenous Times 2025). While such partnerships can provide much-needed funds to an under-represented sector, industry funding can create conflicts of interest (Parker *et al*. 2021).

Pharmaceutical industry sponsorship of health and consumer organizations is well-documented in the literature (Marks 2020, Bero and Parker 2021). However, this is the first study to explore these dynamics within Aboriginal and Torres Strait Islander contexts. Further work is needed to understand the extent of pharmaceutical industry engagement with Aboriginal organizations and to support ACCHOs to navigate industry engagement decisions in ways that safeguard their values, cultural integrity, and quality of care for Aboriginal and Torres Strait Islander communities. Although not specifically raised by participants in our study, recent reports have flagged concerns about increasing pharmaceutical industry promotion of glucagon-like peptide-1 (GLP-1) receptor agonist weight loss medications in Australia (Australian Broadcasting Corporation 2025, Walker *et al*. 2026). This promotion may drive demand for these drugs in ways that restricts access for treating type 2 diabetes (their original application), a condition nearly three times more prevalent in Aboriginal and Torres Strait Islander communities (Hare *et al*. 2022). The marketing practices of the pharmaceutical industry have been documented in the global CDoH literature (Jacob 2018). Given that direct-to-consumer advertising of prescription medications is illegal in Australia, further investigation is needed to understand whether marketing of these drugs is occurring and the implications this may have for Aboriginal and Torres Strait Islander peoples (Hall and Jones 2007, Wutzke *et al*. 2007, Walker *et al*. 2026).

Access to and quality use of medicines through appropriate prescribing, dispensing, and administration is critical in ensuring equitable health outcomes for Aboriginal and Torres Strait Islander peoples. Multiple barriers to quality use of medicines have been identified, including cost, geographical distance to pharmacy services, culturally unsafe care, polypharmacy, and complex medication regimes, all reinforced by an under-resourced and fragmented healthcare system (Rothwell *et al*. 2025). Our findings suggest there may be potential for genuine collaboration between ACCHOs and the pharmacy sector such as those outlined between the National Aboriginal Community Controlled Health Organisation and the PSA. Medicines sector-funded scholarships may further support the development of the Aboriginal pharmacy workforce, provided adequate conflict of interest safeguards are in place. While industry partnerships may offer pathways to culturally safe care, they must be balanced with strategies to manage potential conflicts of interest that risk undermining public trust (Kerridge *et al*. 2005). Strengthening trust in medicines and the pharmacy sector requires Aboriginal and Torres Strait Islander leadership and cultural governance, as highlighted in previous research (Graham *et al*. 2022).

Having Aboriginal and Torres Strait Islander peoples actively leading decisions about how the pharmaceutical industry and pharmacy sector actors operate and engage with issues pertaining to communities is essential for improving the systems, practices, and pathways through which the medicines sector influences health and wellbeing. This includes creating pathways for more Aboriginal pharmacists, supporting community pharmacists to work within ACCHOs as well as ensuring pharmaceutical industry actors engage in genuine collaboration that enables access and distribution of cultural medicines in ways that uphold Indigenous cultural and intellectual property rights.

A key strength of this study lies in its Aboriginal-led design, which centred Aboriginal voices and worldviews through culturally safe methods such as yarning. It is also the first study we are aware of specifically examining the pharmaceutical industry and the pharmacy sector in the Aboriginal and Torres Strait Islander context, filling a critical gap in the CDoH literature. The inclusion of diverse participants across academia, public health, pharmacy, ACCHO, NGO, and government sectors enhanced the breadth and richness of the findings. Additionally, having a large representation of Aboriginal pharmacists ensured the voices of Aboriginal practitioners working in this sector were central to the analysis. Our commitment to ethical research and reporting processes enhanced both methodological and cultural rigour. Our findings were limited by under-representation of perspectives from people in rural and remote Australia, including the Torres Strait, whose experiences of the medicines sector may differ from those living in urban areas. Industry and government actors were also underrepresented in our sample; however, their perspectives are already available in the public domain through policy statements and media releases. Finally, while this study examined commercial systems and practices, the findings are grounded in participant perceptions, which may not fully capture the structural mechanisms through which commercial activities influence health.

## Conclusion

This Aboriginal-led study demonstrates the complex and often contested relationship between the pharmaceutical industry and the pharmacy sector and Aboriginal and Torres Strait Islander health and wellbeing. Our findings reveal how systems and practices within the medicines sector shape access to medicines, Indigenous cultural and intellectual property rights, pharmacy services, and culturally safe care and therefore have the potential to both promote health and to exacerbate inequities. While some medicines sector initiatives show potential, particularly when driven by Aboriginal leadership and grounded in community control, concerns about tokenistic engagement, structural constraints, conflicts of interest and profit-driven models and the political influence of medicines sector actors can undermine Aboriginal and Torres Strait Islander health priorities and may generate unintended health inequities. Policy and practice responses must therefore include transparent governance of industry partnerships, stronger conflict of interest safeguards and Aboriginal and Torres Strait Islander-led decision-making about whether, when, and how commercial actors engage with ACCHOs and cultural knowledge, including traditional medicines. Regulatory pathways for medicine approval and subsidization must be designed to support community access and self-determination rather than enabling commercial exploitation by the pharmaceutical industry. Strengthening culturally safe practices, embedding pharmacy services within ACCHOs and elevating Aboriginal and Torres Strait Islander governance across all aspects of the medicine supply chain are essential steps towards transforming these systems, addressing power imbalances, and advancing health equity. Future Aboriginal-led research should continue to monitor commercial practices and regulatory reforms to ensure they uphold Indigenous peoples’ rights to health equity and self-determination.

## Data Availability

We do not have ethics approval to share our data.
